# Diagnostic value of cone beam computed tomography for root canal morphology assessment – a micro-CT based comparison

**DOI:** 10.1007/s00784-024-05580-y

**Published:** 2024-03-08

**Authors:** Mariana Pires, Jorge N.R. Martins, Mário Rito Pereira, Isabel Vasconcelos, Rui Pereira da Costa, Isabel Duarte, António Ginjeira

**Affiliations:** 1 Faculdade de Medicina Dentária, Universidade de Lisboa, Lisboa, Portugal; 2 Grupo de Investigação em Bioquímica e Biologia Oral, Unidade de Investigação em Ciências Orais e Biomédicas (UICOB), Faculdade de Medicina Dentária, Universidade de Lisboa, Lisboa, Portugal; 3 Centro de Estudo de Medicina Dentária Baseada na Evidência (CEMDBE) - Cochrane Portugal, Faculdade de Medicina Dentária, Universidade de Lisboa, Lisboa, Portugal; 4 Center for Rapid and Sustainable Product Development, Polytechnic Institute of Leiria, Marinha Grande, Portugal; 5 TEMA - Centre for Mechanical Technology and Automation, Department of Mechanical Engineering, University of Aveiro, Aveiro, 3810-193 Portugal; 6 LASI - Intelligent Systems Associate Laboratory, Guimarães, Portugal

**Keywords:** CBCT, Diagnosis, Endodontics, Mandibular molars, Micro-CT, Root canal anatomy

## Abstract

**Objectives:**

The aim of this study was to assess cone beam computed tomography (CBCT) as a root canal anatomy diagnostic tool by comparison with micro-CT gold-standard.

**Materials and methods:**

216 two-rooted mandibular molars were first scanned in a CBCT device (200 μm voxel size) and posteriorly in a micro-CT scanner (19.61 μm). The volumes were sequentially screened to classify main root canal anatomy according to Vertucci classification, and for the presence of lateral canals and apical deltas, in both mesial and distal roots.

**Results:**

Both methods revealed a higher prevalence of Vertucci Type II and IV in the mesial root, and Vertucci Type I in the distal root. The percentage of agreement for main root canal anatomy classification between CBCT and micro-CT scores was high (85.2%).

**Conclusion:**

Sensibility to detect both lateral canals and apical deltas with CBCT was low. These results attest to the fact that minor anatomical changes might be difficult to identify with CBCT imaging, hampering its diagnostic value.

## Introduction

Studies on root canal anatomy have been a constant in the dedicated literature over the years, with an impact on treatment planning and chemo-mechanical preparation procedures´ systematization which allowed maximization of endodontic tretament success. The major change in this type of studies has been largely related to the methods of analysis employed [1]. Although the staining and clearing techniques were once considered the standard, accepted and recommended methodology, with certified scientific value, a more recent trend has been to privilege nondestructive, less invasive and 3-dimensional (3D) assessment tools in both in vitro and in vivo studies [2].

Cone beam computed tomography (CBCT) overcomes the drawbacks of conventional 2-dimensional (2D) radiography, allowing the acquisition and manipulation of 3D images of the same structure or region of interest. Its value in Endodontics is undeniable for laboratory studies but especially in clinical assessments and clinical practice. It is a conservative method that employs a relatively low dose of radiation, especially when small fields of view (FOVs) are selected, and it is quick to perform [3]. Nonetheless, there are still documented limitations that include distortion of the acquired images and variation in image quality between different CBCT scanners [4].

Micro-computed tomography (micro-CT) imaging is a high-resolution radiographic technology that renders sequential 2D images of an object allowing the reconstruction of 3D models (creating 3D rendered volume), and the 2D and 3D morphological data of the root canals [5, 6]. It is, unlike the sectioning and clearing techniques, a non-destructive technique that creates higher quality images than conventional 2D radiographs, and CBCT images [7]. Owing to the higher resolution, micro-CT performs better in identifying and detecting small intricacies of root canal anatomy that may go undetected in CBCT imaging observation [3]. However, it is still a technology limited to extracted teeth, which prevents its application in a clinical context [8].

Mandibular molars - first molars in particular - are considered more prone to undergo endodontic treatment throughout life, probably due to the fact that they are usually the first permanent teeth to erupt, resulting in a higher probability to sustain carious lesions [9]. Its several morphological and anatomical variations have been thoroughly documented [10], including ethnic variability, presence of lateral canals and *isthmi*, and prevalence of morphological alterations such as C-shaped canals [11–13].

Although CBCT has been recognized as a reliable and reproducible method to identify the main root canal anatomy, its lower image resolution, compared to micro-CT imaging, might hamper its ability to detect more complicated anatomical configurations and minor anatomical features [14]. Hence, the goal of the present study is to use micro-CT images as a gold reference to assess the diagnostic value of CBCT in identifying mandibular molars root canal configurations according to Vertucci classification, as well as the presence of lateral canals and apical deltas.

## Materials and methods

### Sample selection and specimens’ preparation

After local research ethics in health committee approval (registration number CE-FMDUL2023BD1), a total of 216 extracted two-rooted mandibular molars were collected from a tooth bank. Previously endodontically treated teeth, or roots presenting with visible cracks, caries lesions, incomplete formation or open apices were excluded. Patients’ age, gender and ethnicity were unknown and were considered irrelevant to the study. The reasons for extractions were unrelated with the present investigation. If any existed, remnants of soft and hard tissue on the surface of the roots were removed using an ultrasonic scaler (P5 Newtron XS; Satelec Acteon, Merignac, France), and the teeth disinfected with a 0.5% chloramine solution and stored in distilled water at 4ºC until the assessment.

### Cone beam computed tomography scanning

All teeth were mounted on a rubber base to allow specimens stabilization, and scanned using a CBCT scanner (Veraview X800, J. Morita Mfg. Corp, Japan) operated at 90 kV and 2 mA, with a field of view of 40 × 40 mm and a voxel size of 200 μm.

### Micro-computed tomography scanning

Posteriorly, the same teeth were also scanned using a Bruker SkyScan 1275 device (Bruker-micro-CT, Kontich, Belgium). The selected scan parameters were: voxel size of 19.61 μm, X-ray tube voltage of 80 kV, X-ray intensity of 125 mA, exposure time of 58 ms, rotation step of 0.5º, 360º rotation, frame averaging of 3, and 1 mm aluminum filter. The micro-CT scans were performed using a specifically designed and validated holder [15].

### Screening method

An equal step-by-step screening methodology was accomplished in both CBCT and micro-CT assessments. Although distinct imaging software were used (i-Dixel One Volume Viewer [Ver.2.6.0, February, 2012, J. Morita Mfg. Corp, Japan] for CBCT, and DataViewer [SkyScan, Bruker micro-CT, Kontich, Belgium] for micro-CT), they allowed equivalent interface and screening protocol. All the relevant variables were assessed by continuous analysis of the axial, sagittal, and coronal views after having their long axis aligned with the ones of the CBCT and micro-CT imaging software. The same operator (MP) was responsible for the evaluation of all teeth. Viewer pre-sets, including angulation or contrast, were adjusted according to individual preference.

**Fig. 1:**
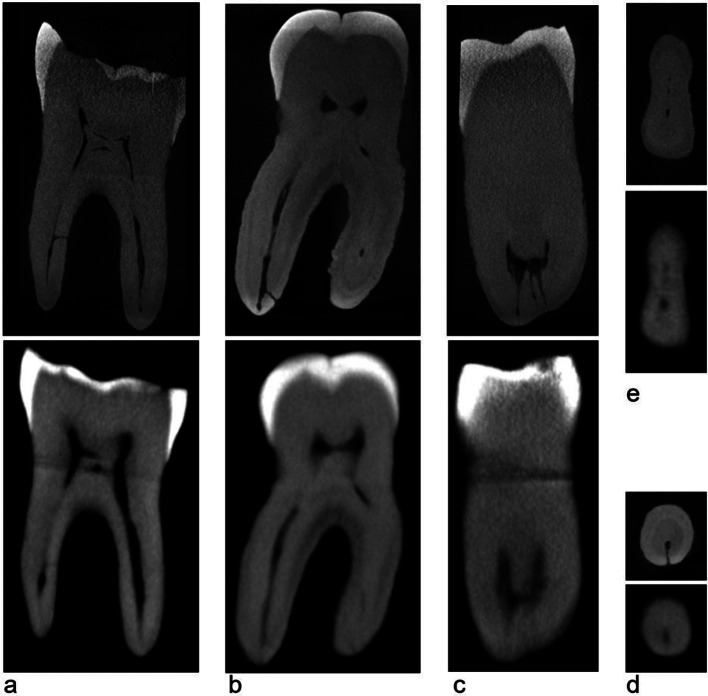
Representative images of specimens showing lateral canals (**a** and **b**) and apical delta (**c**) (sagittal views); and specimens with a canal ramification (**e**) and an apical lateral canal (**d**) (axial views). Micro-CT on top and CBCT on bottom

The three major anatomic features (root canal classification, and lateral canals and apical deltas presence) were assessed for both mesial and distal roots of each one of the 216 teeth (representing a total of 432 evaluated roots). For both CBCT and micro-CT assessments, the analyzed data was recorded according to the following parameters:

- Root canal classification [16], classified into 6 different categories: Vertucci type I (1–1); Vertucci type II (2 − 1); Vertucci type III (1-2-1); Vertucci type IV (2–2); Vertucci type V (1–2); or Others (O). It should be highlighted that for the present research, a canal fusion (merger of 2 canals into 1 single one, for instances type II or type III) was considered even when this confluence was noted in the 2 apical millimeters. However, a canal splitting (1 single canal dividing into 2 canals, for instances type III or type V) was only considered if present coronal to the apical 3-mm level. A splitting within the apical 3 millimeters was considered as an apical anatomic complexity such apical lateral canal or an apical delta, depending on the definition hereby presented.
- Lateral canals (scored as presence/absence) which were defined as any branch of the main pulp canal that communicated with the external root surface [17] (Fig. 1).
- Apical deltas (scored as presence/absence) which were defined as a pulp configuration in which the main canal splits into multiple apical accessory canals [17]. In the present study, the term “multiple” was defined as the presence of at least two apical ramifications (besides de the main root canal) in the apical 3 millimeters. One single ramification (besides the main root canal) was accounted as an apical lateral canal and accounted as such (Fig. 1).

### Intra-rater reliability assessment

An intra-rater reliability assessment was conducted by evaluating twice the initial 18 teeth (8.6% of the overall sample) by both CBCT and micro-CT imaging. The same observer conducted both analysis with a two week interval and in the second assessment was blinded regarding the first one results. Vertucci configuration, lateral canals and apical deltas outcomes were compared using the Cohen kappa test and the results expressed in percentages and asymptotic standard error (SPSS v22.0 for Windows, SPSS Inc., Chicago, IL, USA).

### Statistical analysis

The Vertucci types, lateral canals and apical deltas presence outcomes were expressed in total counts and proportions for both CBCT and micro-CT imaging. While the assessment of the CBCT as a possible diagnostic tool was determined by comparing its outcomes with the ones from the micro-CT gold standard reference. The CBCT sensibility, positive and negative predictive values, specificity, accuracy and percentage of agreement were determined and presented in percentages.

## Results

Six teeth were excluded due to root fracture during their mounting on the micro-CT holder. The intra-rater kappa coefficient of agreement between both evaluations showed a substantial agreement [18] on the Vertucci classification (CBCT: 67.5% with an asymptotic standard error ± 9.6%; micro-CT: 71.6% ± 9.1%), presence of lateral canals (micro-CT: 71.0% ± 11.5%) and presence of apical deltas (CBCT: 65.4% ± 32.0%; micro-CT: 78.6% ± 20.6%) assessments. While the presence of lateral canals screened by CBCT had a no agreement (19.5% ± 15.7%) result [18].

The root canal configuration assessment, conducted by both CBCT and micro-CT, showed a higher percentage of Vertucci type II and IV in the mesial root, while in the distal one Vertucci type I was the most common. The anatomic characteristics of the global sample (210 mandibular molars) is summarized in Table 1.

**Table 1:** Overview of the anatomic characteristics of the mandibular molars included in the study assessed by both CBCT and micro-CT

Anatomic feature	CBCT		Micro-CT	
	Mesial (n = 210)	Distal (n = 210)	Mesial (n = 210)	Distal (n = 210)
Root canal configuration according to Vertucci classification				
Vertucci I	8.1% (17)	75.7% (159)	10.0% (21)	80.0% (168)
Vertucci II	42.4% (89)	14.3% (30)	41.4% (87)	13.8% (29)
Vertucci III	5.2% (11)	2.3% (5)	4.3% (9)	1.4% (3)
Vertucci IV	38.1% (80)	3.3% (7)	37.6% (79)	1.9% (4)
Vertucci V	3.8% (8)	2.9% (6)	3.3% (7)	1.9% (4)
Other	2.3% (5)	1.4% (3)	3.3% (7)	0.9% (2)
Accessory anatomy				
Lateral Canal	44.3% (93)	23.8% (50)	58.6% (123)	31.4% (66)
Apical Delta	1.9% (4)	2.3% (5)	12.9% (27)	7.1% (15)

The sensibility of the CBCT to identify the several Vertucci configuration types was higher for type I (89.4%), type II (82.8%) and type IV (90.4%), which represented 80.9% (340/420) of all configurations. A lower sensibility score was observed in type III (58.3%), type V (54.5%) and “others” (55.5%). As for the specificity and accuracy, a high score was noted in all Vertucci configuration types with the lower limits observed in Vertucci type II (specificity: 92.4%; accuracy: 89.8%). The percentage of agreement between CBCT and Micro-CT scores was also high (85.2%) (Table 2).

Regarding the presence of lateral canals, the CBCT imaging specificity (81.4%), accuracy (68.6%), and the percentage of agreement between the CBCT and the reference scores (68.6%), were lower when compared to the Vertucci configuration result. Additionally, the CBCT imaging showed a low sensibility (52.9%) in detecting lateral canals (Table 2).

As for the apical delta presence assessment, despite the high CBCT imaging accuracy (89.8%) and percentage of agreement with the Micro-CT imaging (89.8%), the CBCT imaging sensibility to detect these anatomic features was very low (9.5%) (Table 2).

**Table 2:** – CBCT imaging sensitivity, positive and negative predictive value, specificity, accuracy and agreement (with the reference) according to specific anatomic features

Factor	Sensitivity	Positive Predictive Value	Negative Predictive Value	Specificity	Accuracy	Percentage Agreement
**Mesial Root**						
Vertucci I	76.2% (16/21)	94.1% (16/17)	97.4% (188/193)	99.5% (188/189)	97.1% (204/210)	83.3%
Vertucci II	87.4% (76/87)	85.4% (76/89)	90.9% (110/121)	89.4% (110/123)	88.6% (186/210)	
Vertucci III	44.4% (4/9)	36.4% (4/11)	97.5% (194/199)	96.5% (194/201)	94.3% (198/210)	
Vertucci IV	91.1% (72/79)	90.0% (72/80)	94.6% (123/130)	93.9% (123/131)	92.8% (195/210)	
Vertucci V	57.1% (4/7)	50.0% (4/8)	98.5% (199/202)	98.0% (199/203)	96.2% (202/210)	
Other	42.9% (3/7)	60.0% (3/5)	98.0% (201/205)	99.0% (201/203)	97.1% (204/210)	
Lateral Canal	54.5% (67/123)	72.0% (67/93)	52.1% (61/117)	70.1% (61/87)	60.9% (128/210)	60.9%
Apical Delta	11.1% (3/27)	75.0% (3/4)	88.3% (182/206)	99.5% (182/183)	88.1% (185/210)	88.1%
**Distal Root**						
Vertucci I	91.1% (153/168)	96.2% (153/159)	70.6% (36/51)	85.7% (36/42)	90.0% (189/210)	87.1%
Vertucci II	69.0% (20/29)	66.7% (20/30)	94.4% (171/180)	94.5% (171/181)	90.1% (191/210)	
Vertucci III	100% (3/3)	60.0% (3/5)	100% (205/205)	99.0% (205/207)	99.0% (208/210)	
Vertucci IV	75.0% (3/4)	42.8% (3/7)	99.5% (202/203)	98.1% (202/206)	97.6% (205/210)	
Vertucci V	50.0% (2/4)	33.3% (2/6)	99.0% (202/204)	98.1% (202/206)	97.1% (204/210)	
Other	100% (2/2)	66.7% (2/3)	100% (207/207)	99.5% (207/208)	99.5% (209/210)	
Lateral Canal	50.0% (33/66)	66.0% (33/50)	79.4% (127/160)	88.2% (127/144)	76.2% (160/210)	76.2%
Apical Delta	6.7% (1/15)	20.0% (1/5)	93.2% (191/205)	97.9% (191/195)	91.4% (192/210)	91.4%
Overall						
Vertucci I	89.4% (169/189)	96.0% (169/176)	91.8% (224/244)	96.9% (224/231)	93.6% (393/420)	85.2%
Vertucci II	82.8% (96/116)	80.6% (96/119)	93.4% (281/301)	92.4% (281/304)	89.8% (377/420)	
Vertucci III	58.3% (7/12)	38.9% (7/18)	98.8% (399/404)	97.8% (399/408)	96.7% (406/420)	
Vertucci IV	90.4% (75/83)	86.2% (75/87)	97.6% (325/333)	96.4% (325/337)	95.3% (400/420)	
Vertucci V	54.5% (6/11)	42.8% (6/14)	98.8% (401/406)	98.0% (401/409)	96.7% (406/420)	
Other	55.5% (5/9)	62.5% (5/8)	99.0% (408/412)	99.3% (408/411)	98.3% (413/420)	
Lateral Canal	52.9% (100/189)	69.9% (100/143)	67.8% (188/277)	81.4% (188/231)	68.6% (288/420)	68.6%
Apical Delta	9.5% (4/42)	44.4% (4/9)	90.8% (373/411)	98.7% (373/378)	89.8% (377/420)	89.8%

## Discussion

Knowledge of root canal anatomy is the first step in planning for a thorough root canal treatment or retreatment. Besides the main root canal considerations, smaller intricacies, including lateral canals and apical deltas, must be considered due to their possible impact in treatment outcome [5, 19].

The advent of high-resolution 3D image acquisition methods has changed the paradigm in anatomical studies in Endodontics. Micro-CT, a non-destructive high resolution 3D imaging method is now considered and used as the gold-standard for evaluation of internal root canal anatomy [14]. However, clinical application is currently not possible due to its high radiation dose. CBCT also allows a high-resolution 3D image acquisition whilst applying a lower radiation dose, making it applicable in vivo, which has contributed to its elevation in both clinical and laboratorial studies [20]. Several publications, with different study designs, have employed CBCT to conduct an analysis of mandibular molar anatomy, but most lack comparison to a reference that is as close to reality as possible [21]. It is, therefore, and especially from a clinical point of view, of interest to compare and validate CBCT imaging as a diagnostic tool, using Micro-CT as reference [22].

**Fig. 2:**
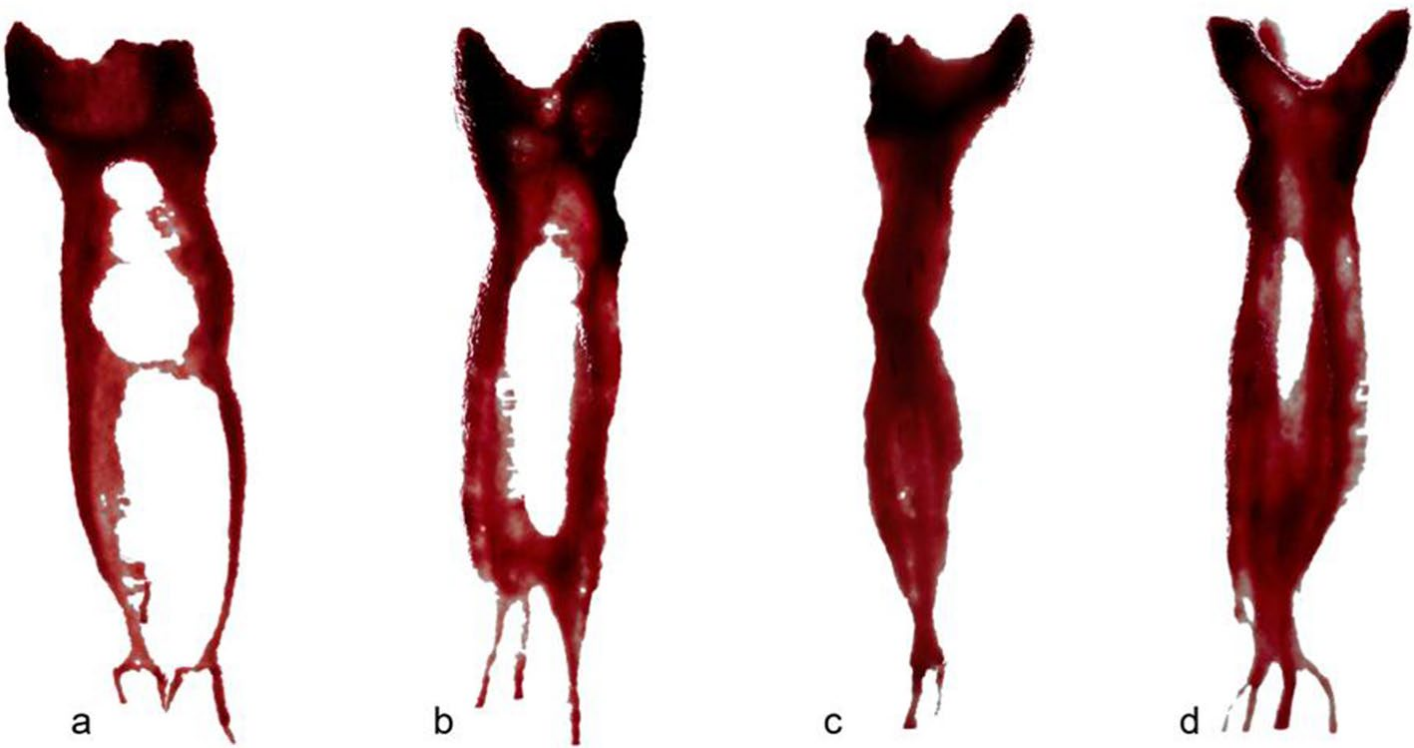
Micro-CT representative 3D reconstruction of mesial canals Vertucci type IV with lateral canal (**a**); mesial canals Vertucci type II with apical delta (**b**); distal canal Vertucci type I with lateral canal (**c**); distal canal Vertucci type II with apical delta (**d**). The shown details are presently not available in CBCT imaging and possible to be used in clinical practice

Our investigation revealed a higher prevalence of Vertucci type II and type IV morphologies in mesial roots of extracted mandibular molars, and Vertucci type I in the distal roots of the same specimens, which is in accordance with previous reports [21, 23], and additionally showing a high level of agreement between the two diagnostic tools (85,2%) on their identification. Representative 3D reconstructions of the most prevalent anatomies are shown in Fig. 2 taking advantage of a micro-CT resolution not yet available for CBCT imaging. CBCT has been used in both in vivo and ex vivo studies to assess main root canal morphology and, aligned with this investigation, shows globally a good match with the micro-CT gold-standard imaging, validating this method as reliable for main root canal anatomy classification. Both values of specificity and accuracy were considerably high. However, it is important to point-out that the higher the complexity of the root canal anatomy (Types III, V, others), the lower the values of sensibility obtained, meaning that more intricate root canal morphologies are more easily misdiagnosed in a CBCT exam.

Contrary to main root canal morphology assessment, the sensibility of CBCT to detect lateral canals and apical deltas was low and very low, respectively (Table 2; Fig. 1). The literature available is considerably scarcer when referring to the use of CBCT to detect such types of minor anatomic variations. A previous study on the reliability of CBCTs to detect lateral canals before and after root canal treatment analyzed 43 extracted premolars and concluded that CBCT was an unreliable tool for diagnosis of lateral canals. Despite employing micro-CT in the methodology, the scans were only performed after root canal filling and were not used for the purpose of comparison reference [14]. Another investigation aimed at comparing CBCT to periapical radiographs in identifying apical deltas in extracted premolars, using micro-CT as a reference, concluded that CBCT imaging failed at detecting 65% of existing apical deltas in the analyzed specimens [24]. The quality of the images rendered from a CBCT scan can be influenced by several technical and clinical aspects [14]. Particularly in the field of Endodontics, smaller FOVs and voxel sizes such as the ones selected in the present study are recommended since these allow higher spatial resolution [25]. However, high spatial resolution also results in higher noise in the final images, resulting in higher difficulty to identify finer structures [24]. Clinically, previous studies indicate that lateral and accessory apical foramina – and, necessarily, the lumen of the lateral canals and apical ramifications leading to these portals of exist – can be 3 times more constrict than the main apical foramen [26]. An exact protocol determining an optimal equilibrium between voxel size and noise reduction is still to be determined when it comes to identifying minor anatomical structures [27].

Despite the corroborated value of CBCT in Endodontics, its limitations must be acknowledged and considered in adherence to the as low as reasonably achievable principle (ALARA) [3]. The present investigation validates CBCT as a reliable method for main root canal morphology identification and classification in mandibular molars, while raising concerns regarding its applicability when the aim is identifying minor anatomical variations. Further investigations should focus on how to optimize CBCT image acquisition to more accurately allow identification of these intricacies [28]. Also, despite the large clinical sample employed, there was no record of patient data from which the sample was obtained, nor distinction was made between first, second and third molars, thus limiting extrapolation of results. Another limitation of the present assessment is that the screening of the data was performed by a single operator. Future investigations should include more than one observer, thus allowing inter-rater agreement calculation for an increased value of the methodology. Clinically, and since such intricacies are likely to be left untouched by instrumentation, the focus should be on maximizing the effect of chemical debridement with adjunctive techniques such as ultrasonic activation of irrigants [29].

## Conclusions

CBCT constitutes a reliable diagnostic tool for understanding the major anatomic configuration of mandibular molars. However, it is not accurate in identifying minor anatomical structures, such as lateral canals and apical deltas. Care must be taken when prescribing this additional radiographic exam, bearing in mind its current limitations.
